# P300 Spatiotemporal Prior-Based Transformer-CNN for Auxiliary Diagnosis of PTSD

**DOI:** 10.3390/brainsci15101124

**Published:** 2025-10-19

**Authors:** Lize Tan, Hao Fang, Peng Ding, Fan Wang, Yuanyuan Wei, Yunfa Fu

**Affiliations:** 1 Faculty of Information Engineering and Automation, Kunming University of Science and Technology, Kunming 650500, China; tanlize0807@gmail.com (L.T.); auasr_schorr@163.com (P.D.); fanwang991@gmail.com (F.W.); 2 Brain Cognition and Brain-Computer Intelligence Integration Group, Kunming University of Science and Technology, Kunming 650500, China; 3 Faculty of Art and Design, Wuhan Institute of Technology, Wuhan 430205, China; fanghao@wit.edu.cn (H.F.); a360901@163.com (Y.W.)

**Keywords:** brain–computer interface, PTSD, P300, transformer, CNN

## Abstract

**Objectives:** To address the challenges of subjectivity, misdiagnosis and underdiagnosis in post-traumatic stress disorder (PTSD), this study proposes an objective auxiliary diagnostic method based on P300 signals. Existing studies largely rely on conventional P300 features, lacking the systematic integration of event-related potential (ERP) priors and facing limitations in spatiotemporal feature modeling. **Methods:** Using common spatiotemporal pattern (CSTP) analysis and quantitative evaluation, we revealed significant spatiotemporal differences in P300 signals between PTSD patients and healthy controls. ERP prior information was then extracted and integrated into a hybrid architecture combining transformer encoders and a convolutional neural network (CNN), enabling joint modeling of long-range temporal dependencies and local spatial patterns. **Results:** The proposed P300 spatiotemporal transformer-CNN (P300-STTCNet) achieved a classification accuracy of 93.37% in distinguishing PTSD from healthy controls, markedly outperforming traditional approaches. **Conclusions:** Significant spatiotemporal differences in P300 signals exist between PTSD and healthy control groups. The P300-STTCNet model effectively captures PTSD-related spatiotemporal features, demonstrating strong potential for electroencephalogram-based objective auxiliary diagnosis.

## 1. Introduction

Post-traumatic stress disorder (PTSD) is a complex psychiatric condition triggered by exposure to severe trauma or stressful events. Its core symptoms include intrusive memories, avoidance behaviors, negative alterations in mood and cognition, and persistent hyperarousal [1,2]. These symptoms are often accompanied by pronounced physiological activation, such as increased heart rate, sleep disturbances, and exaggerated startle responses, which can significantly impair social functioning and quality of life [3].

Early diagnosis of PTSD is crucial for timely intervention and treatment [4], as it can reduce the risk of symptom chronicity and mitigate comorbid mental disorders and impairments in social functioning [5,6]. Although systematic diagnostic criteria have been established, current procedures still heavily rely on subjective assessments and clinical interviews, which are susceptible to individual cognitive biases and evaluation variability, leading to prominent issues of misdiagnosis and underdiagnosis [7]. Therefore, developing efficient and objective strategies for early diagnosis is of significant value for optimizing patient outcomes and alleviating public health burdens.

With advances in neuroimaging and electrophysiological techniques, research based on objective biomarkers has provided greater accuracy and reliability in the diagnosis of psychiatric disorders. Electroencephalography (EEG), with its high temporal resolution, non-invasiveness, and repeatability, has become a vital tool for investigating neural function and aiding diagnosis in mental health research [8]. Event-related potential (ERP), derived from EEG recordings, are neurophysiological signals that reflect the brain’s time-locked responses to specific sensory, cognitive, or motor events. Among ERP components, the P300 is a key indicator, closely associated with attention allocation and cognitive processing, and has therefore received considerable attention in neurophysiological studies of PTSD [9].

In recent years, there has been growing interest in elucidating the neural mechanisms underlying PTSD. Studies have demonstrated that individuals with PTSD exhibit significant abnormalities in P300 responses during cognitive tasks, primarily characterized by reduced amplitudes and prolonged latencies. Changes in P300 amplitude and latency reflect individual differences in information processing efficiency and the allocation of attention to target stimuli [10]. These abnormalities are closely associated with dysfunctions in key brain regions, including the prefrontal cortex, amygdala, and hippocampus [11], suggesting that P300 possesses potential biomarker value in the spatiotemporal domain.

Existing research on neural mechanisms provides an important theoretical foundation for the objective identification of PTSD. Some studies have primarily relied on single features from specific electrodes, such as peak amplitude or mean latency, to quantify PTSD [12,13,14]. Although these metrics can partially reflect trauma-related cognitive and neural activity changes [15], their informational content is limited, making it difficult to comprehensively reveal inter-regional brain interactions and the dynamic characteristics of the P300, thereby constraining the sensitivity and specificity of classification. Notably, these neurophysiologically based findings still provide critical prior ERP information, such as key electrode channels and latency time windows, which can serve as important prior constraints for deep learning models and offer theoretical guidance for the objective diagnostic identification of PTSD.

Against the backdrop of rapid advances in artificial intelligence, P300 features combined with machine learning methods have been widely applied for PTSD recognition [16]. For example, Shim et al. [17] achieved a classification accuracy of 73.33% using the mean P300 amplitude feature combined with support vector machine (SVM); Terpou et al. [18] extracted P300 temporal features and combined them with SVM to reach 76% accuracy; and Kim et al. [19] employed source covariance matrices with Riemannian geometry, achieving 75% accuracy. Qu et al. [20] utilized a convolutional neural network—long short-term memory (CNN-LSTM) framework on differential entropy features to achieve 80% accuracy, while Beykmohammadi et al. [21] combined eyes-closed resting-state EEG with wavelet transform and transfer learning using a VGG16 network for deep learning-based PTSD classification. Although these studies have advanced the diagnostic and recognition capabilities for PTSD, limitations remain: most approaches rely on handcrafted features, and to our knowledge, no study has systematically integrated ERP prior information in the analysis of PTSD P300 signals, leaving relevant neural activity patterns and mechanisms insufficiently explored. Moreover, existing methods still face challenges in modeling the complex, high-dimensional spatiotemporal dependencies of EEG, particularly the P300 component.

Based on the aforementioned limitations, this study proposes a transformer-CNN deep learning model, P300 spatiotemporal transformer-CNN (P300-STTCNet), which integrates ERP priors to systematically identify abnormal spatiotemporal patterns of P300 signals in PTSD patients compared with healthy controls (HC). In this model, the transformer encoder, leveraging the multi-head self-attention mechanism, demonstrates a significant advantage in modeling global dependencies within time series, while the CNN, as an efficient feature extractor, effectively captures the spatial distribution characteristics of EEG signals. By integrating these two components complementarily, the model achieves synergistic spatiotemporal modeling, thereby overcoming the CNN’s limitations in capturing long-range dependencies due to its restricted receptive field. Furthermore, ERP prior information is extracted using the common spatiotemporal pattern (CSTP) method and systematically incorporated into the model, enabling more precise and objective auxiliary diagnosis of PTSD.

## 2. Materials and Methods

### 2.1. Participants

This study recruited 15 patients diagnosed with PTSD at the Department of Psychiatry, the First Affiliated Hospital of Kunming Medical University, between March 2024 and June 2025. All patients were diagnosed by clinical psychiatrists according to the criteria of the Diagnostic and Statistical Manual of Mental Disorders, Fourth Edition, Text Revision (DSM-IV-TR, American Psychiatric Association, 2011), in conjunction with the PTSD Checklist (PCL) scale. During the study, one patient withdrew due to dizziness and nausea from discomfort with the EEG cap, and two patients declined participation for personal reasons. Additionally, 12 HC participants were recruited. Ultimately, complete datasets were successfully collected from 12 PTSD patients and 12 HCs. All participants provided written informed consent prior to the experiment. The study was conducted in strict accordance with the Declaration of Helsinki and approved by the Ethics Committee of Kunming University of Science and Technology Hospital (Approval No. KMUST-MEC-056).

To ensure consistency of experimental conditions, all participants completed the experiment in a ventilated, well-lit, and quiet laboratory environment free from external disturbances. The experiments were conducted during daytime, and participants maintained their regular diet and daily routines prior to the experiment to minimize the potential influence of physiological state variations on EEG signals.

### 2.2. Experimental Paradigm

**Figure 1:**
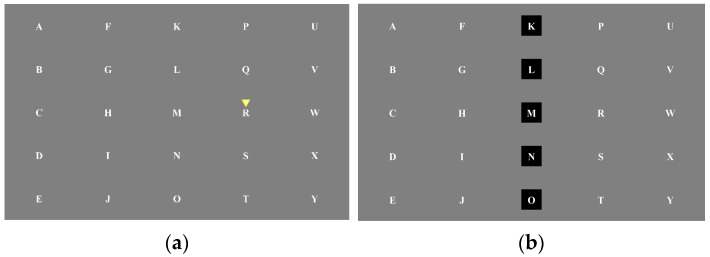
P300 experimental paradigm. (**a**) The target character is indicated by a yellow inverted triangle; (**b**) Random flashing of rows and columns.

The experiment employed a classic 5 × 5 single-target P300 character matrix paradigm, as illustrated in Figure 1. The study consisted of one session, with each session comprising two runs. Each run was divided into two blocks, and each block contained 25 trials. During each trial, participants were required to respond to a target stimulus, which was a letter indicated by an inverted triangle, presented for 1 s. To ensure sustained attention, the black rows and columns in the stimulus matrix randomly changed every 0.5 s. The total duration of the experiment for each participant was approximately 13 min, comprising two runs with a brief inter-run break to mitigate potential fatigue effects on the experimental outcomes.

### 2.3. Data Acquisition

**Figure 2:**
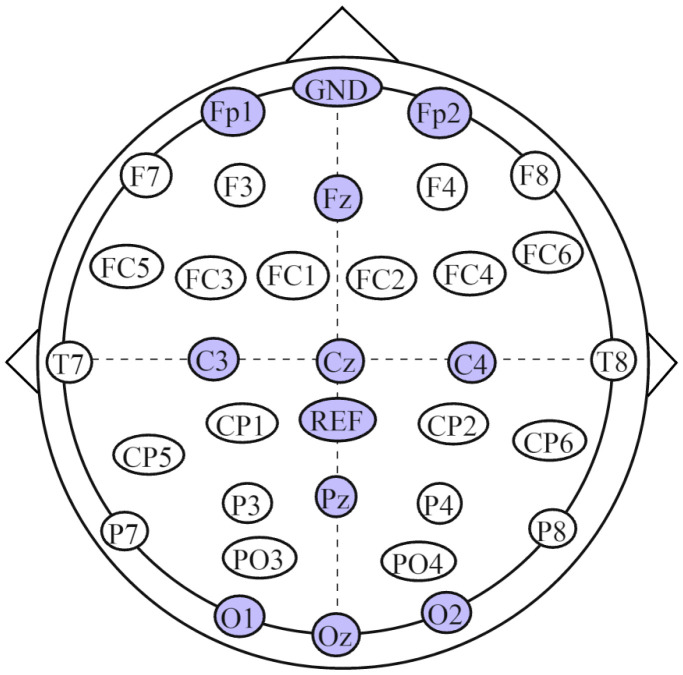
P300 electrode layout.

EEG data were acquired using a NeuSen W wireless digital 32-channel EEG amplifier (Borycon Technology Co., Ltd., Changzhou, China) at a sampling rate of 1000 Hz. Electrode placement followed the international 10–20 system. The ground electrode was positioned at FPz, and the reference electrode at CPz. The electrodes used in this study included Fp1, Fp2, Fz, C3, Cz, C4, Pz, O1, Oz, and O2, with the detailed electrode layout shown in Figure 2.

### 2.4. Preprocessing

EEG data collected during the P300 experiment are typically susceptible to noise. The preprocessing procedures in this study included the following steps: (1) selection of 10 target channels (Fp1, Fp2, Fz, C3, Cz, C4, Pz, O1, Oz, O2) from the original 32-channel data, along with extraction of the corresponding labels; (2) The EEG data were average-referenced using the EEGLAB toolbox and band-pass filtered between 0.1 and 30 Hz to remove frequency components unrelated to the P300; (3) Ten independent components were extracted using the fast independent component analysis (FastICA) algorithm implemented in EEGLAB, and 1–2 artifact-related components were removed based on automatic identification results; (4) segmentation of the preprocessed data into epochs, with each stimulus onset set to 0 ms, capturing a time window from −100 ms to 600 ms to encompass the full P300 response; (5) baseline correction using the −100 ms to 0 ms interval to eliminate baseline shifts, followed by downsampling to 250 Hz.

### 2.5. Spatiotemporal Difference Analysis

In the spatiotemporal feature extraction of multi-channel EEG, traditional methods such as common spatial pattern and xDAWN primarily focus on spatial filtering, with relatively limited modeling of temporal structures [22]. Methods based on Riemannian geometry or tensor decomposition can capture multidimensional information to some extent, but they still exhibit limitations in feature discriminability and stability [23]. In contrast, the CSTP approach explicitly enhances the differences between the two classes in their spatiotemporal distributions by jointly modeling spatial and temporal covariance matrices, making it more suitable for capturing the concurrent abnormalities of P300 in both latency and spatial distribution. Its objective function can be expressed as:

(1) $$\underset{W,V}{\max}\frac{tr(W^{T}\Sigma_{1}W\cdot V^{T}R_{1}V)}{tr(W^{T}\Sigma_{2}W\cdot V^{T}R_{2}V)}$$

Here, W denotes the spatial projection matrix, V the temporal projection matrix, and $\Sigma_{1}$, $\Sigma_{2}$ and $R_{1}$, $R_{2}$ represent the spatial and temporal covariance matrices of the two classes of signals, respectively. To solve this objective function, an alternating optimization strategy is typically employed. W and V are iteratively computed through eigendecomposition, with the eigenvectors corresponding to the largest eigenvalues selected at each iteration.

By jointly applying spatial and temporal filtering, CSTP can effectively capture the most discriminative components in the spatiotemporal domain. Let a multichannel signal sample be represented as *X* ∈ *R^C^*^×^*^T^*, where *C* denotes the number of channels and *T* denotes the number of time points. CSTP sequentially maps the raw signals into a low-dimensional spatiotemporal feature space through the spatial and temporal projection matrices *W* and *V*:

(2) $$Z=W^{T}X_{i}V$$

Here, Z represents the signal matrix after spatiotemporal projection. The specific temporal and spatial energy features are calculated as follows:

(3) $$f_{space}\left(c\right)=log\left(\frac{var(Z_{i}\left(m,:\right))}{\sum_{m^{\prime }=1}^{M}var(Z_{i}\left(m^{\prime },:\right))}\right)$$

(4) $$f_{time}\left(t\right)=log\left(\frac{var(Z_{i}\left(:,k\right))}{\sum_{k^{\prime }=1}^{K}var(Z_{i}\left(:,k^{\prime }\right))}\right)$$

Here, $f_{space}\left(c\right)$ and $f_{time}\left(t\right)$ represent the normalized log-variance features of the signal at channel c and time point t, respectively, thereby enabling discriminative representation of spatiotemporal features.

The spatiotemporal features extracted by CSTP are typically high-dimensional, encompassing both spatial and temporal information. Although high-dimensional features can comprehensively represent data variability, they may introduce challenges such as increased computational complexity, information redundancy, and susceptibility to noise in subsequent analyses and visualization. To address these issues, principal component analysis (PCA) was applied for dimensionality reduction, preserving the most representative features while reducing complexity. PCA was performed separately on the raw data and CSTP-extracted features, and the resulting components were effectively visualized to highlight inter-group feature differences.

To further quantify the differences in spatiotemporal features between the PTSD and HC groups, trial-averaging was employed for the comparative analysis of the P300 signals. Group differences were examined across all EEG channels, and topographical maps were generated to visualize spatial distribution differences. Additionally, peak amplitudes and latencies within the 250–500 ms time window were systematically analyzed to compare P300 characteristics between the PTSD and HC groups.

In the statistical analysis, to evaluate the spatiotemporal differences in P300 signals between the two groups, independent-sample *t*-tests were first conducted on the mean amplitudes of the averaged P300 signals across all EEG channels. Bonferroni correction was applied to the results of multiple comparisons to control the false positive rate. Statistical significance was determined by multiplying the original *p*-value of each channel by the number of tests and comparing it to α = 0.05. In addition, independent-sample *t*-tests were also performed on the peak amplitude and latency data within the 250–500 ms time window, with *p*-values adjusted using Bonferroni correction.

### 2.6. P300-STTCNet Model

**Figure 3:**
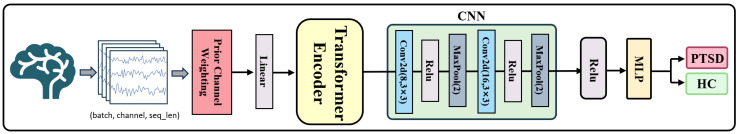
P300-STTCNet model architecture. Note: The “Prior Channel Weighting” module applies prior bias weighting to EEG channels; ReLU (rectified linear unit); MLP (multilayer perceptron).

Based on the analysis of spatiotemporal differences in P300 signals between the PTSD and HC groups, channel-wise prior biases and attention priors from ERP information were further extracted. Building on this, we propose a P300 spatiotemporal feature model, P300-STTCNet, which integrates ERP priors with a hybrid transformer-CNN architecture. The model framework is illustrated in Figure 3.

In the proposed model, the preprocessed P300 signals are first processed through channel-wise prior bias weighting. Specifically, a learnable weight parameter is assigned to each input channel. During initialization, based on the analysis of spatiotemporal differences in P300 signals, higher initial weights are assigned to the Fz, C3, Cz, and C4 channels, incorporating spatial ERP priors to emphasize their critical role in P300 recognition for PTSD patients. These weight parameters are adaptively updated during model training, enabling dynamic modeling and optimization of the relative contributions of different channels.

**Figure 4:**
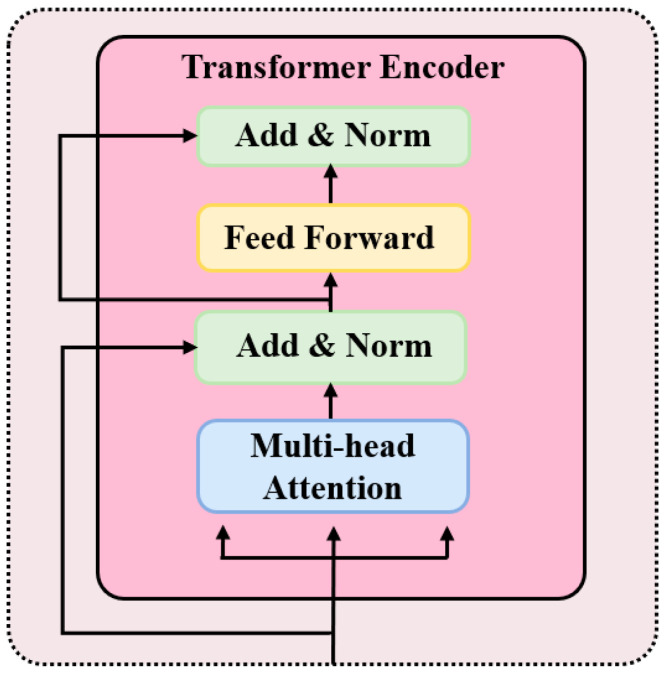
Transformer encoder. Note: “Add and Norm” denotes the residual connection and layer normalization.

Subsequently, the signals are mapped through a fully connected layer and fed into a four-layer stacked transformer encoder, enhancing the representation of key temporal features in the P300 signals. The structure of the transformer encoder module is illustrated in Figure 4 and is primarily composed of a multi-head attention (MHA) mechanism and a feed forward network (FFN). Each encoder layer contains these two sublayers and employs residual connections and layer normalization to improve both training efficiency and model stability.

**Figure 5:**
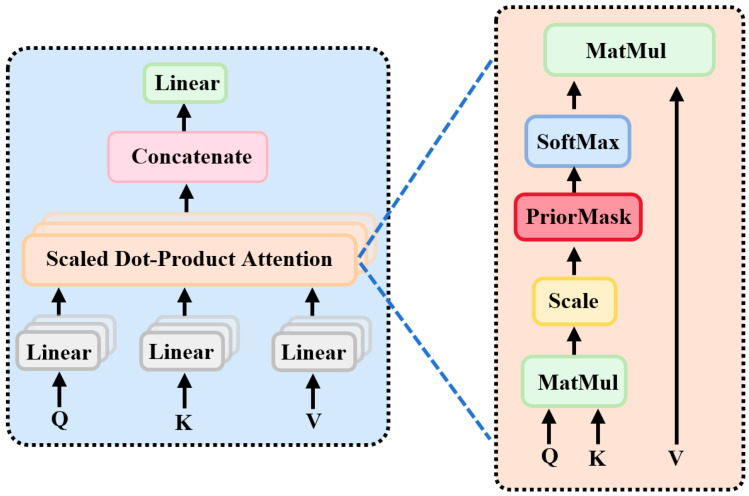
Multi-head attention mechanism. Note: “PriorMask” represents the prior attention mask derived from the temporal ERP prior information of the P300 signal.

The MHA mechanism is the core component of the transformer encoder, designed to capture global dependencies across different positions in the input sequence, as illustrated in Figure 5. MHA consists of multiple parallel attention heads, each performing independent linear projections of the input sequence into query (Q), key (K), and value (V) representations, followed by feature interaction through scaled dot-product attention. Specifically, each head computes the dot product between Q and K to measure positional relevance, scales the result to stabilize gradients, incorporates a prior attention mask (PriorMask), and applies the softmax function to obtain attention weights. The weighted sum with V yields the head output. Finally, the outputs from all heads are concatenated and linearly projected to form the final MHA representation.

Let the input sequence be $S\in R^{n\times d}$, where n denotes the sequence length and, d the dimensionality of each input. MHA processes the input data in parallel through multiple independent attention heads, and the outputs of these heads are concatenated. The internal structure of each attention head is illustrated on the right side of Figure 5. The computation within each self-attention head can be expressed by the following equations:

(5) $$Q=SW_{Q}$$

(6) $$K=SW_{K}$$

(7) $$V=SW_{V\;}$$

Here, $W_{Q}$, $W_{K}$,and $W_{V}$ are learnable weight matrices corresponding to the linear transformations of the query Q, key K, and value V, respectively.

Next, the dot products between all Q and K vectors are computed and scaled by $\sqrt{d_{k}}$. On this basis, a prior attention mask is introduced to incorporate temporal ERP priors of the P300 signal. Specifically, based on latency analyses of P300 signals from the PTSD and HC groups, a critical time window (250–450 ms) is identified, and a prior attention mask is applied to these intervals. Positions corresponding to this time window in the attention matrix are assigned positive weights, guiding the Transformer encoder to focus on key temporal ERP features of the P300 signal during attention computation. The results are normalized using a softmax function to obtain the attention weights, which are then applied to V to generate the final attention output. The corresponding formulation is given in Equation (8):

(8) $$Attention\left(Q,K,V\right)=softmax\left(\frac{QK^{T}}{\sqrt{d_{k}}}+PriorMask\right)V$$

Here, $K^{T}$ denotes the transpose of K, $d_{k}$ is the dimensionality of the key vectors, and PriorMask represents the prior attention mask.

MHA aggregates the outputs of multiple heads by concatenation. The output of the i-th attention head, ${head}_{i}$, is computed as follows:

(9) $${head}_{i}=Attention\left(QW_{i}^{Q},KW_{i}^{K},VW_{i}^{V}\right)$$

Here, $W_{i}^{Q}$, $W_{i}^{K}$, and $W_{i}^{V}$ are the learnable weight matrices corresponding to the query, key, and value for the i-th attention head, respectively.

The final output of the MHA is obtained through the linear transformation matrix $W^{O}$:

(10) $$MultiHead\left(Q,K,V\right)=Concat\left({head}_{1},\ldots ,{head}_{h}\right)W^{O}$$

After temporal modeling, the extracted features are fed into the CNN layers. The first convolutional layer employs 16 two-dimensional kernels of size 3 × 3, while the second layer uses 32 kernels of the same size. The kernel size and the number of convolutional channels were determined based on preliminary experimental results to achieve an optimal balance between model complexity and classification performance. These convolutional operations enable the model to effectively capture local spatial patterns, and the incorporation of ReLU activation functions introduces nonlinear mappings to enhance feature representation. Subsequently, pooling layers are applied to reduce feature dimensionality, thereby lowering computational complexity and mitigating overfitting. Finally, the high-dimensional features are input into a MLP and mapped to the output space to generate the final classification results.

We employed a subject-level 4-fold cross-validation strategy. All subjects were randomly divided into four groups following a class-balanced principle to ensure representative category distribution in each fold. In each iteration, data from three groups were used for training, while the remaining group served as the test set. This process was repeated four times so that each group was used as the test set once. For baseline comparison, the spatiotemporal features of the P300 extracted via the CSTP method were input into a SVM classifier. The SVM adopted a radial basis function kernel with a penalty coefficient of C = 0.1, and the training protocol was kept consistent with that of the P300-STTCNet model.

## 3. Results

### 3.1. Difference Analysis

**Figure 6:**
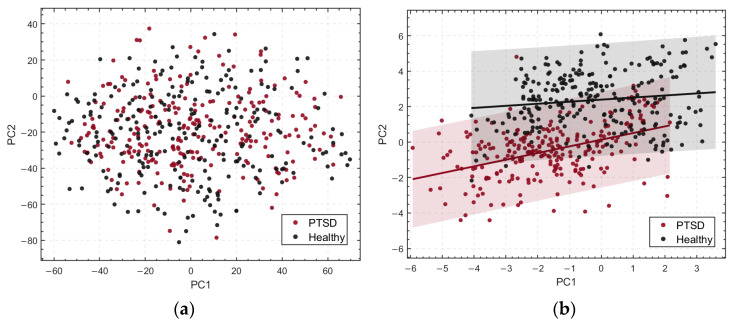
Differences between the PTSD group and the HC group on principal components PC1 and PC2. (**a**) Distribution of Raw Features; (**b**) Distribution of CSTP-Extracted Features, shaded regions indicate 95% prediction intervals.

The raw EEG data and the P300 spatiotemporal features extracted via CSTP were reduced in dimensionality using PCA. For visualization purposes, the first two principal components (PC1 and PC2) were selected to generate scatter plots illustrating the distribution differences between the PTSD and HC groups, as shown in Figure 6. In the plots, PC1 and PC2 serve as the horizontal and vertical axes, respectively, with different colors distinguishing the groups (red representing the PTSD group and black representing the HC group). For the raw data, PC1 and PC2 accounted for approximately 38.1% and 26.7% of the total variance, respectively, while for the CSTP-extracted P300 spatiotemporal features, PC1 and PC2 explained about 35.5% and 23.4% of the total variance. The principal component axes are annotated in the CSTP feature visualization to more clearly illustrate the distributional differences between groups.

In the PCA-reduced representation of the raw data, samples from the PTSD and HC groups exhibited substantial overlap, indicating low discriminability of the original features between the two groups. In contrast, for the CSTP-extracted spatiotemporal features, the distributions of PTSD and HC samples in the PC1–PC2 space showed noticeable separation, with significantly improved inter-group differentiation. This suggests that CSTP-extracted features more effectively capture differences between the groups, although some overlap remains and intra-group feature distributions are relatively dispersed. The separation along the principal component axes further validates the ability of CSTP features to distinguish spatiotemporal differences between groups.

After trial-wise averaging of the P300 signals from all subjects in both the PTSD and HC groups, independent sample *t*-tests were conducted across ten EEG electrode channels. Bonferroni correction was applied to the results of multiple comparisons to assess the statistical differences between the two groups at each channel. The statistical outcomes, including *p*-values, t-values, effect sizes (Cohen’s d), and significance levels for each channel, are summarized in Table 1.

Based on the *p*-value analysis, the Fz, C3, Cz, and C4 channels exhibited significant inter-group differences (p_m_ < 0.05), indicating notable amplitude differences in P300 signals between the PTSD and HC groups at these channels. Specifically, at Fz, the t-value (t_m_) was −2.886, corresponding to a Cohen’s d (d_m_) of −1.228; at C3, t_m_ = −1.988 and d_m_ = −0.846; at Cz, t_m_ = 1.278 and d_m_ = 0.544; and at C4, t_m_ = −1.778 with d_m_ = −0.758. In contrast, the Fp1, Fp2, Pz, O1, Oz, and O2 channels showed a p_m_ greater than 0.05, failing to reach statistical significance, suggesting no notable differences in P300 signals between the groups at these locations.

**Figure 7:**
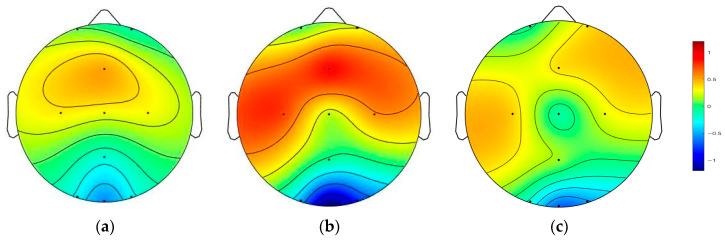
Grand average topographic maps of the mean P300 signals across channels and their corresponding amplitude ranges. (**a**) Topographic map of the PTSD group; (**b**) Topographic map of the HC group; and (**c**) The difference topographic map (HC minus PTSD).

By calculating the average P300 signals across all participants in the PTSD and HC groups and visualizing the results using topographical maps, inter-group differences at the major electrode sites were further demonstrated, as shown in Figure 7. The left panel displays the average topographic map of the PTSD group, the middle panel shows that of the HC group, and the right panel presents the difference map (HC minus PTSD), with a color bar indicating the range of values. Clear inter-group differences were observed at the Fz, C3, and C4 channels, where P300 signal amplitudes differed markedly between the PTSD and HC groups.

To further investigate the differences in P300 waveform characteristics between the PTSD and HC groups, independent-sample *t*-tests were conducted on the peak amplitude and latency data within the 250–500 ms time window. As shown in Table 1, the peak amplitude analysis revealed significant inter-group differences at the Fz (p_a_ = 0.0021) and C3 (p_a_ = 0.0114) electrodes, indicating notable amplitude differences between the PTSD and HC groups at these sites. The latency analysis showed significant differences at Fp2 (p_l_ = 0.001), Fz (p_l_ = 0.006), C3 (p_l_ = 0.043), C4 (p_l_ = 0.036), Pz (p_l_ = 0.026), O1 (p_l_ = 0.015), and O2 (p_l_ = 0.015). These results indicate that the PTSD group exhibits significant differences from the HC group in both P300 amplitude and latency across multiple electrode sites.

**Figure 8:**
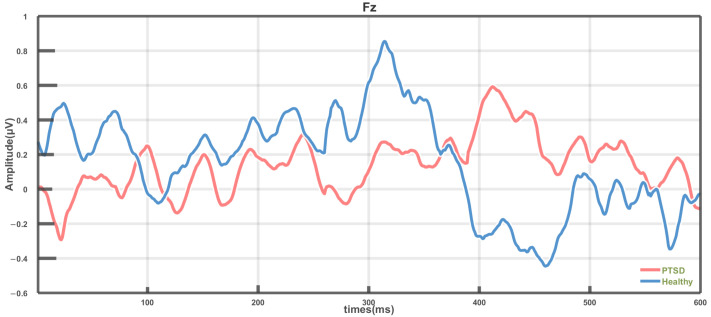
Average P300 waveforms at the Fz electrode.

After trial-averaging, independent-samples *t*-tests were performed for each electrode channel as well as for peak amplitudes and latencies. The Fz electrode consistently demonstrated significant inter-group differences. To visually represent the P300 waveform characteristics at this key site, the average P300 waveforms at Fz were plotted. Figure 8 illustrates the mean P300 waveforms for the PTSD and HC groups at the Fz electrode, with the HC group represented by the blue curve and the PTSD group by the red curve.

Significant differences were observed between the PTSD and HC groups in both amplitude and latency of the P300 component at the Fz electrode. Specifically, the PTSD group exhibited overall lower P300 amplitudes compared with the HC group, with a generally flatter waveform trend. Within the 250–500 ms time window, the HC group displayed a more pronounced positive peak, whereas the PTSD group showed a relatively attenuated positive peak. Regarding latency, the P300 peak occurred at approximately 312 ms in the HC group, while it was delayed to around 415 ms in the PTSD group.

### 3.2. Model Performance

**Figure 9:**
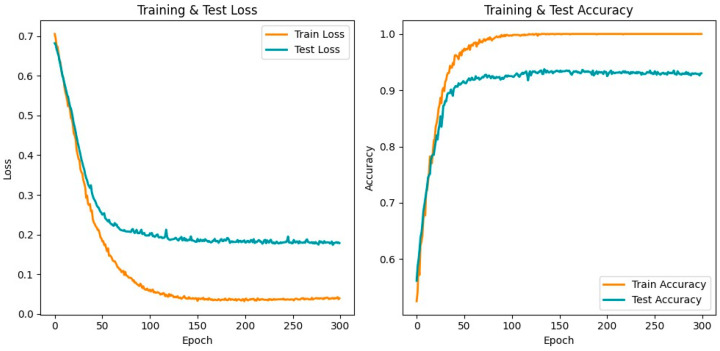
Training results of the P300-STTCNet model for PTSD vs. HC classification.

The P300-STTCNet model achieved an average classification accuracy of 93.37% (95% CI: 90.92–95.82%) on the test set for distinguishing PTSD from HC, with the detailed training results shown in Figure 9. The model’s average classification performance on the test set was as follows: precision 94.48% (95% CI: 92.32–96.64%), recall 92.46% (95% CI: 91.39–93.53%), and F1 score 93.43% (95% CI: 91.97–94.89%). The left panel of Figure 9 depicts the loss curves for both the training and test sets, showing a gradual decrease in loss that stabilizes as training progresses. The right panel illustrates the trends in the training and test accuracies, with the yellow line representing the training set and the blue line representing the test set.

**Figure 10:**
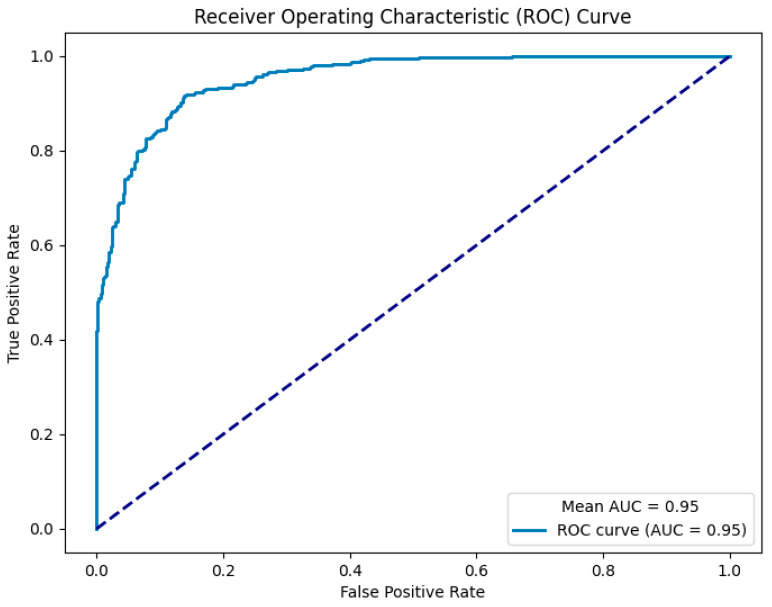
ROC curve of the P300-STTCNet model. Note: Dashed diagonal line indicates the random classifier baseline (AUC = 0.5).

The proposed P300-STTCNet model achieved an Area Under the Receiver Operating Characteristic Curve (AUC) of 0.95 in the classification task, as shown in Figure 10.

**Figure 11:**
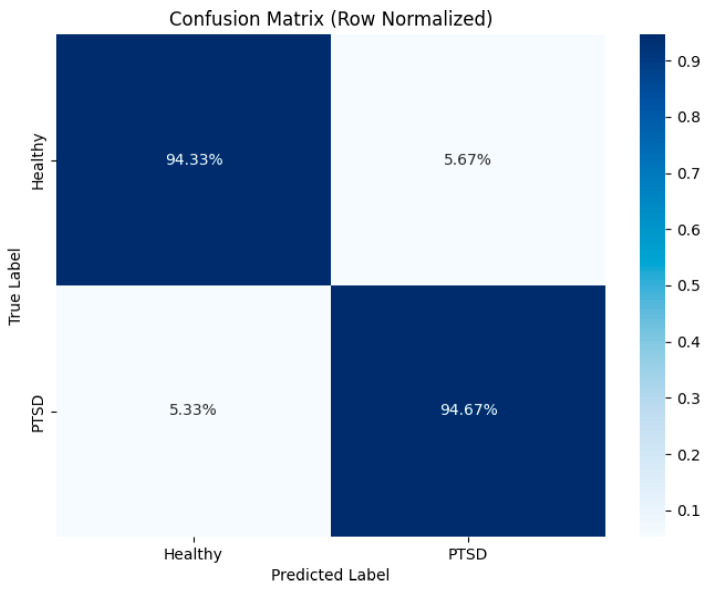
Confusion matrix of the P300-STTCNet model for PTSD vs. HC classification.Note: Dashed diagonal line indicates the random classifier baseline (AUC = 0.5).

Figure 11 presents the confusion matrix of the P300-STTCNet model for the PTSD versus HC classification task, with row-wise normalization applied. The vertical axis represents the true labels, and the horizontal axis corresponds to the predicted labels, while the color bar on the right indicates the normalized proportion range. This confusion matrix provides a visual assessment of the model’s classification performance, where diagonal elements denote correctly classified proportions and off-diagonal elements represent misclassification rates.

The proposed P300-STTCNet model was compared with existing studies. Detailed results are presented in Table 2, which summarizes the classification accuracy of our method in comparison with previous approaches.

Compared with existing P300-based classification methods, the proposed P300-STTCNet model demonstrated relative advantages in the auxiliary diagnosis of PTSD. In this study, a baseline comparison was also conducted using SVM classification on P300 spatiotemporal features extracted via CSTP based on spatiotemporal difference analysis, achieving an accuracy of 84.6%, which outperforms most existing approaches. Furthermore, by integrating ERP priors and incorporating both a Transformer encoder and CNN to capture the spatiotemporal dependencies of P300 signals, the P300-STTCNet model further improved classification accuracy to 93.37%. These results indicate that the proposed model not only provides enhanced representational capacity for P300 signal modeling but also exhibits potential applicability and translational value in clinical PTSD auxiliary diagnosis.

## 4. Discussion

This study systematically investigated differences in P300 waveforms between PTSD and HC groups by comparing their spatiotemporal features. Results based on CSTP feature extraction and PCA dimensionality reduction demonstrated that, compared with the raw data, the CSTP-extracted spatiotemporal features exhibited significantly enhanced the inter-group separation along the first two principal components (PC1 and PC2). This indicates that the method effectively differentiates P300 feature patterns between PTSD and HC groups and suggests potential involvement of abnormal activity in the specific brain regions associated with cognitive processing and neurophysiological functions.

Although CSTP features exhibit strong discriminative power for inter-group differences, some overlap between groups and within-group variability remain, likely due to individual differences in EEG signals and the limitation of visualizing only the first two principal components. Future studies may consider incorporating additional principal components or combining alternative dimensionality reduction techniques to further improve feature separability and interpretability.

The topographic map results further demonstrated a significant reduction in P300 amplitude at frontal and central electrode sites in the PTSD group. Independent-sample *t*-tests with Bonferroni correction indicated statistically significant differences between the PTSD and HC groups in both P300 amplitude and latency at the Fz, C3, Cz, and C4 electrodes. These findings are consistent with Shim et al. [10], who reported reduced P300 amplitudes and prolonged latencies in PTSD patients, suggesting that P300 abnormalities may represent a neurophysiological hallmark of PTSD and potentially aid in distinguishing PTSD from other psychiatric disorders such as depression.

Further analysis revealed that, particularly at the Fz electrode, the PTSD group exhibited more pronounced differences in both amplitude and latency, with overall lower and flatter P300 waveforms. Within the 250–500 ms time window, the HC group displayed a more typical positive peak, whereas the PTSD group showed markedly attenuated amplitude. Additionally, the P300 peak latency in the PTSD group was delayed by approximately 103 ms compared to the HC group (HC: 312 ms; PTSD: 415 ms), indicating delayed or less efficient cognitive processing within this time window and reflecting potential deficits in information processing [24]. These results not only deepen the understanding of the neurocognitive mechanisms underlying PTSD but also support the utility of P300 as a potential neurophysiological biomarker, providing a biologically meaningful and interpretable approach for objective auxiliary diagnosis.

Building on the aforementioned spatiotemporal difference analysis, the proposed P300-STTCNet model was trained on preprocessed P300 signals and achieved a high classification accuracy. The area under the ROC curve reached 0.95, indicating that the model possesses high discriminative power and stability in distinguishing between PTSD and HC individuals. The model incorporates ERP prior information during channel-weight initialization to emphasize the contribution of key electrodes, and a prior attention mask was designed for the 250–450 ms critical time window within the attention mechanism, enabling the Transformer encoder to focus on core P300 features.

Previous literature [25] has highlighted that SVMs are the most widely used traditional classifiers in PTSD auxiliary diagnosis studies, while some deep learning models, such as EEGNet and transfer learning-based VGG16, have also demonstrated relatively high classification performance. However, these approaches largely rely on frequency-domain features of whole-brain EEG or other generalized metrics, lacking targeted modeling of ERP components such as the P300. In contrast, this study focuses on the P300 as a core neurophysiological feature and proposes the P300-STTCNet model, which integrates ERP prior information to more effectively capture the spatiotemporal characteristics of P300 signals. In the PTSD auxiliary diagnosis task, the model achieved a classification accuracy of 93.37%, significantly outperforming most traditional SVM-based methods and demonstrating strong discriminative performance and clinical application potential for PTSD identification.

However, this study has several limitations. First, although the spatiotemporal features of P300 were fully considered, frequency-domain analyses were not incorporated. Previous studies have demonstrated abnormalities in the EEG frequency domain of PTSD patients [26], suggesting that future research could explore the contribution of frequency-domain features to classification performance.

Moreover, the current study’s model does not account for individual differences. Future work could incorporate subject-specific features to develop personalized modeling approaches, thereby enhancing the model’s generalizability. Additionally, subgroup analyses could be introduced in subsequent studies to enable the model to discriminate among different subpopulations. Given the limited sample size and the inherent difficulty of data collection in this study, it can be considered a pilot study, and the statistical power may be somewhat constrained. Therefore, validation on larger-scale datasets is required in the future to strengthen the robustness and generalizability of the conclusions.

Furthermore, this study only analyzed P300 signals and ERP priors collected from a single experimental session, without evaluating cross-session reproducibility or feature stability. Considering that EEG signals may exhibit considerable within-subject variability across sessions, the robustness of the extracted features remains to be further verified. Future research could perform multi-session assessments to evaluate reproducibility and to confirm the model’s effectiveness in identifying stable neurophysiological markers.

Finally, although P300 is closely linked to cognitive processing, this study did not integrate other physiological or behavioral measures (e.g., fMRI or cognitive task performance) for cross-validation. Future research could employ multimodal data fusion to further elucidate the impact of PTSD on neurophysiological function, thereby enabling more comprehensive objective assessment and diagnosis.

## 5. Conclusions

This study compared P300 signal features between PTSD and HC groups, revealing significant spatiotemporal differences in individuals with PTSD, primarily manifested as reduced amplitudes and delayed latencies, from which ERP priors were extracted. Based on these findings, a novel P300-STTCNet model was proposed to efficiently capture both temporal dependencies and spatial distribution patterns of P300 signals for the automated classification of PTSD and HC participants. The model innovatively incorporates ERP prior information into a hybrid Transformer-CNN architecture, extracting discriminative feature representations from long-range temporal dependencies and local spatial patterns, respectively. Experimental results demonstrated that P300-STTCNet significantly outperformed conventional methods in terms of classification accuracy, precision, and F1-score. Compared with the models relying solely on amplitude or latency features, P300-STTCNet exhibited superior performance, highlighting its potential for auxiliary PTSD diagnosis and objective assessment of EEG signals.

## Figures and Tables

**Table 1:** Statistical comparison of the mean amplitude, peak amplitude, and latency of P300 at each electrode between the PTSD and HC groups. Note: p_m_ denotes the *p*-value of the independent-sample *t*-test for mean amplitude between PTSD and HC groups; t_m_ represents the corresponding t-value; Cohen’s d_m_ indicates the effect size (Cohen’s d) for mean amplitude; p_a_ is the *p*-value for peak amplitude; and p_l_ is the *p*-value for latency.

Channel	p_m_	t_m_	Cohen’s d_m_	p_a_	p_l_
Fp1	0.09	0.686	0.292	0.084	0.135
Fp2	0.055	−1.173	−0.500	0.062	0.001
Fz	0.005	−2.886	−1.228	0.002	0.006
C3	0.019	−1.988	−0.846	0.011	0.043
Cz	0.048	1.278	0.544	0.065	0.071
C4	0.02	−1.778	−0.758	0.053	0.036
Pz	0.059	0.944	0.402	0.072	0.026
O1	0.17	0.341	0.145	0.089	0.015
Oz	0.54	0.104	0.044	0.291	0.078
O2	0.35	0.262	0.112	0.157	0.015

**Table 2:** Comparison of Classification Accuracies Between Previous Studies and the Proposed Method.

Study	Features	Classifier	Accuracy
Shim et al. [10]	P300 amplitude, latency, and source	SVM	80%
Shim et al. [17]	P300 mean amplitude	SVM	73.33%
Terpou et al. [18]	P300 spatiotemporal features	SVM	76%
Kim et al. [19]	Source covariance	Riemannian geometry	75.24%
Ours	CSTP-extracted P300 spatiotemporal features	SVM	84.6%
	P300-STTCNet		93.37%

## Data Availability

The data presented in this study are available on request from the corresponding author due to patient privacy and ethical restrictions. The informed consent permits use only within the research team, and public sharing would violate ethics approval regulations.
